# Rapid colorimetric antimicrobial susceptibilities direct from positive blood culture for Gram-negative bacteria

**DOI:** 10.1128/spectrum.02147-25

**Published:** 2025-09-19

**Authors:** Prakash C. Khanal, Joseph C. Richardson, Katherine G. Richardson, Gregory L. Damhorst, Owen J. Oertell, Alexandra Filbrun, Eileen M. Burd, Robert M. Dickson

**Affiliations:** 1 School of Chemistry & Biochemistry and Institute for Bioengineering and Bioscience, Georgia Institute of Technology 1372, Atlanta, Georgia, USA; 2 Division of Infectious Diseases, Department of Medicine, Emory University School of Medicine 12239, Atlanta, Georgia, USA; 3 Department of Pathology and Laboratory Medicine, Emory University School of Medicine 12239, Atlanta, Georgia, USA; University at Albany, Albany, New York, USA

**Keywords:** susceptibility testing, colorimetric, bloodstream infections, diagnostics, machine learning, optical instrumentation

## Abstract

Bloodstream infections (BSIs) have become increasingly challenging to treat due to emerging antimicrobial resistance (AMR). As rapid administration of appropriate antimicrobials is crucial to positive patient outcomes, clinical alternatives to slow phenotypic antimicrobial susceptibility testing (AST) must be developed. We developed an automated real-time, colorimetric assay (ChroMIC) that phenotypically determines minimum inhibitory concentrations (MICs) directly from positive blood culture. Gram-negative bacteria within positive blood cultures from 83 patients were directly analyzed with ChroMIC. Both ChroMIC MICs and the much slower VITEK 2 MICs for each of seven antibiotics were compared to broth microdilution (BMD). Direct-from-blood-culture ChroMIC MICs were obtained without the ~18-hour growth and isolation steps needed to obtain both VITEK 2 and BMD MICs. ChroMIC assays yielded >90% categorical and essential agreement within 7 h from the start of the experiment and within 4 h of initial growth detection. ChroMIC minor, major, and very major errors were all comparable to, or in the case of VMEs, better than, those from VITEK 2 ASTs—the latter being performed only after an additional ~18-hour subculturing delay. As these patient blood samples were run in parallel with VITEK 2 MICs, a retrospective impact analysis on patient care suggests that ChroMIC could have facilitated faster therapeutic decision-making in nearly all cases, saving an average of >35 and >43 h for antimicrobial escalation and de-escalation, respectively, compared to VITEK 2.

**IMPORTANCE:**

The often sluggish pace of phenotypic antimicrobial susceptibility testing (AST), relative to sepsis progression, limits flexibility in altering patient treatment. We report a new direct-from-blood culture phenotypic AST that delivers excellent results within ~7 h of blood culture positivity. This rapid and accurate determination of optimal bloodstream infection treatment was compared in a retrospective study on patient blood cultures that flagged positive for Gram-negative pathogens. Although standard clinical methods were used to guide treatment, our testing was performed in parallel and could have enabled correction of the treatment course ~40 h earlier than was actually performed. Once clinically implementable, such innovative, low-labor, automated, and accurate susceptibility determinations hold great potential for improving patient outcomes and lowering overall healthcare costs.

## INTRODUCTION

Often leading to sepsis, bloodstream infections (BSIs) are a major cause of global mortality and morbidity (1, 2), with ~677,000 cases and ~94,000 deaths annually in the USA alone (3). Rapid, appropriate BSI treatment is challenged by the need to amplify bacterial loads through 24–72 h blood culture to confirm infection (4, 5), with further ~18 and >30 h delays to identify the pathogen and determine susceptibility, respectively (6–8). BSIs caused by highly resistant bacteria that are not controlled by initial empiric treatments pose a significant threat (9, 10). Thus, after pathogen growth is detected in a blood culture, determining susceptibility becomes the time-limiting treatment step. Broad resistance is particularly problematic in Gram-negative bacteria as they account for >50% of BSIs (11, 12), but their susceptibility profiles are less readily inferred from genetic (13) or other available rapid tests (14). The severity and rapid onset of sepsis due to BSIs force reliance on empiric treatments, even when highly accurate but often too slow and expensive automated antimicrobial susceptibility testings (ASTs) are available (15). Coupled with the need for prior subculturing to isolate pure bacteria, the long time-to-result, high cost, and relatively high skilled labor demands of automated AST instrumentation preclude its use outside of the wealthiest hospital environments (15–17).

Primary and secondary BSIs are a major cause of sepsis, for which time to appropriate treatment is the primary determinant of patient survival. Each hour of delay in initiating appropriate treatment increases the incidence of death by as much as 7.6% during septic shock (18). Gold standard phenotypic ASTs uniquely determine susceptibility irrespective of bacterial resistance mechanisms (19), but their long turnaround times mandate empiric treatment be administered rapidly, often resulting in inappropriate antimicrobial selection (18, 20). Molecular methods, such as polymerase chain reaction (PCR), ePlex, and BioFire FilmArray, enable rapid bacterial identification and detection of specific genes (13, 21). Despite this, their utility is constrained by the limited number of genes they can identify, potentially overlooking newly emerging resistances (21), and they cannot discriminate between viable and non-viable organisms (22). Additionally, genotypic tests may not accurately reflect the phenotypic outcomes, particularly in the case of highly adaptive Gram-negative bacteria (21, 23), and they do not yield quantitative minimum inhibitory concentration (MIC) values (13). Consequently, phenotypic methods continued to be the gold standard method for AST determination.

Blood culture is essential to increase bacterial densities from the initial ~10 CFU/mL to achieve appropriate inoculum sizes of ~5 × 10^5^ CFU/mL. Although obtaining pure colonies from positive blood culture is essential for many commercially available systems, the vast majority of BSIs are monomicrobial, allowing for the creation of an AST directly from positive blood cultures and eliminating the lengthy bacterial isolation step. While some progress on direct from blood culture ASTs has been made, accuracies and costs may not yet meet the needed benchmarks for clinical implementation (24). If sufficiently accurate, easily implementable, and cost-effective, such testing could significantly attenuate BSI-related morbidity and mortality (25), better control antimicrobial resistance proliferation, and drastically improve patient outcomes. Such methods could simultaneously lower patient and overall healthcare costs, as sepsis treatment in the USA alone accounts for total hospital costs exceeding $24 billion annually (26). To address the unmet clinical need, we have developed a rapid colorimetric AST (ChroMIC) for susceptibility determination directly from positive blood cultures, which has potential to be developed into cost-effective, low-labor technology that can be implemented in both low- and high-resource clinical settings.

In this proof-of-concept study, we tested aerobic, monomicrobial Gram-negative positive blood culture samples against seven antibiotics to rapidly determine MICs while avoiding lengthy subculturing steps. ChroMIC assays utilize visual color changes coupled with computer vision and machine learning algorithms to register bacterial growth under antibiotic challenge. Bypassing the subculturing step, ChroMIC provides an inexpensive, automated, rapid alternative to clinically utilize phenotypic susceptibility testing that requires long subculturing steps. In a parallel, retrospective study, we compared both ChroMIC and VITEK 2 ASTs on positive blood cultures from 83 patients, gaging the accuracy of each vs. BMD and the potential for faster ChroMIC to impact patient care and outcomes.

## MATERIALS AND METHODS

### Preparation of antibiotic panels

**Fig 1:**
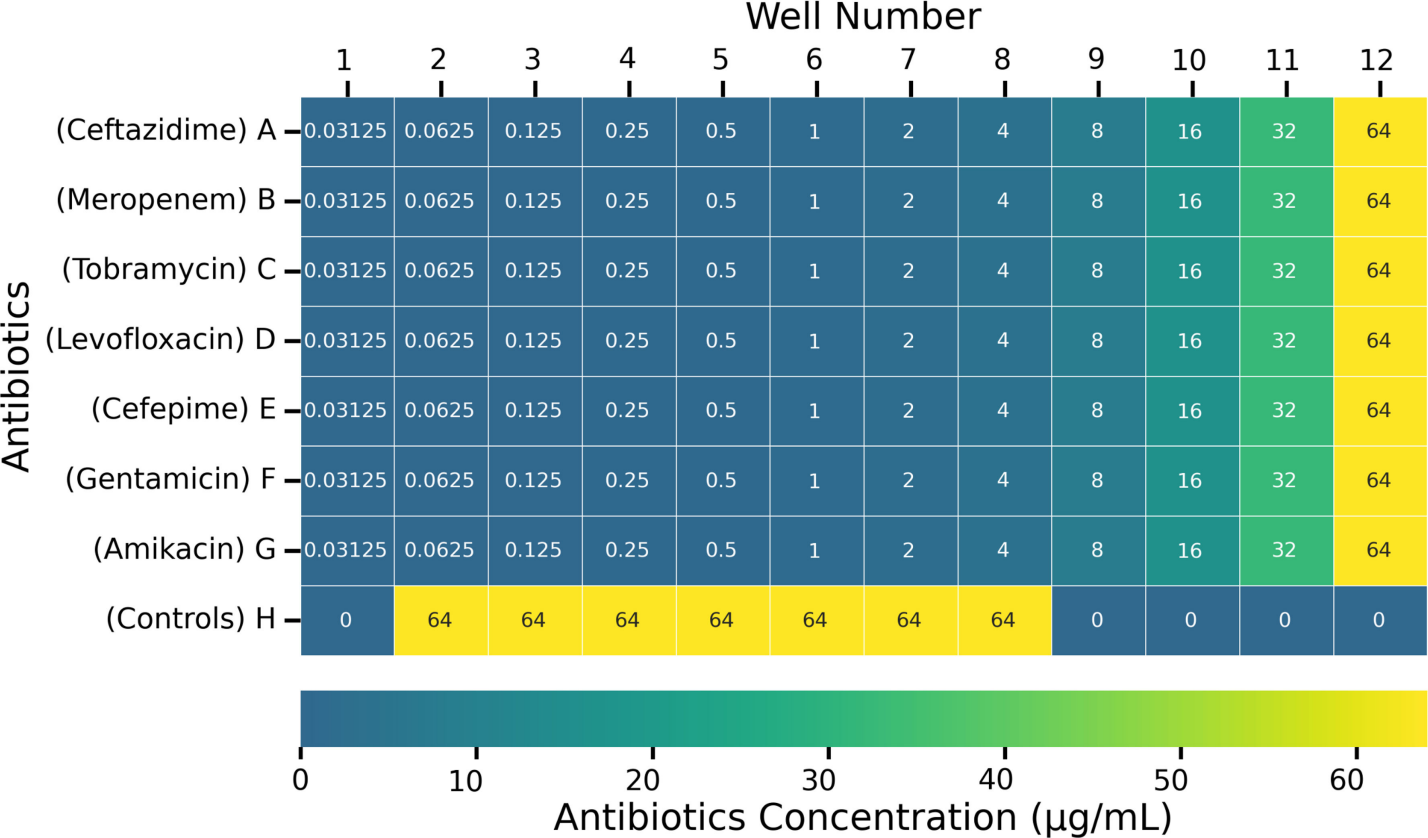
Heat map showing schematic layout of antibiotic panel used for both ChroMIC and BMD assays. Wells H1, H9, and H10 are media-only negative controls. Wells H11 and H12 are no-antibiotic positive controls. Wells H2 through H8 are negative controls containing only media, contrast, and 64 µg/mL of amikacin, gentamicin, cefepime, levofloxacin, tobramycin, meropenem, or ceftazidime, respectively.

Antibiotic panels (seven antibiotics total: ceftazidime, meropenem, tobramycin, levofloxacin, cefepime, gentamicin, and amikacin, Table S1) were prepared by serial two-fold dilutions along each row of 96-well plates following CLSI guidelines (27). Each well of the pre-prepared panels contained 100 µL of solution at 2×the final desired concentration. Upon adding an additional 100 µL of sample to each well (below), the final antibiotic concentration ranged from 0.03125 µg/mL to 64 µg/mL with a final volume of 200 µL in each well (Fig. 1).

### Preparation of contrast medium

Sterile human whole blood (ZenBio, Research Triangle Park, NC) was stored at 4°C and was used within two weeks. The sterility of purchased human whole blood was confirmed by plating on LB agar plates and incubating at 37°C for 24 h. For use in the medium, whole blood was diluted in sterile cation-adjusted Mueller-Hinton broth (CAMHB; BD Biosciences, San Jose, CA) medium (10% vol/vol). Bacterial respiration and growth have been shown to alter the oxy/deoxy-hemoglobin ratio, leading to a distinct bright red to dark red color change (28, 29). We utilize this color change (Fig. S1) to determine the growth or no growth in each well through automated colorimetric analysis.

### ChroMIC assays directly from positive blood culture

Aliquots from positive blood culture bottles were initially diluted 500-fold in CAMHB medium by dispensing 20 µL of positive blood culture into 10 mL of freshly prepared contrast media and gently vortexing for ~10 s. Then, 100 µL of this solution (corresponding to 0.2 µL positive culture) was dispensed into each well of a 96-well plate antibiotic panel to yield the final desired antibiotic concentrations. After the addition of diluted blood culture, final antibiotic concentrations ranged from 0.03125 µg/mL to 64 µg/mL along each row, giving a final 1,000-fold dilution of each original blood culture. Once dispensed, the 96-well plates were covered with a sterile sealing film (VWR International, Radnor, PA), and the assay was initiated by placing in an incubator at 37°C. A schematic of the panel layout with final antibiotic concentrations is shown in Fig. 1. ChroMIC ASTs were performed in parallel with VITEK 2 and compared (blinded) against BMD. Each blood culture was also plated to retrospectively determine bacterial inoculum size corresponding to our ultimate 1,000× dilution.

### Instrument design for ChroMIC assay

Each sealed 96-well microtiter plate was positioned in an incubator (37°C), approximately 6 cm above a computer-controlled, color, 12-megapixel charge-coupled device (CCD) camera (Raspberry Pi HQ camera/Raspberry Pi 4B computer). A low-distortion wide-angle lens (Commonlands, Part number: CIL034-F2.7-M12IP660, wide angle, no distortion, IP67 3.2 mm lens with 87^o^ field of view and an IR cutoff filter) was used to image microtiter plates from below, with white LED illumination from above. Each computer controlled up to four cameras simultaneously with user-written software collecting images once every 15 minutes for 18 h. Each image within these 73-image sequences is analyzed in real time. Computer vision was used to identify the wells, and color space analyses accurately identified perceptible color changes (see next section).

### Contrast mechanism and color analysis

While color change upon bacterial growth in blood-containing media and corresponding MICs can be determined visually, we extracted the intensities from each of the red, green, and blue channels of the color CCD camera used to monitor growth. Intensities from a 40 × 40 pixel area near the central region of each well were extracted and averaged. The three-dimensional RGB values were treated as a vector, and dimensions were reduced to the top two principal color components through principal components analysis. The top principal components typically accounted for >97% of the variance. To discriminate between wells with or without bacterial growth, we utilized a support vector machine (SVM) classifier in the principal color component space. All codes were written in Python 3.11 using scikit-learn packages.

Negative (no-growth) controls included wells without patient sample added, as well as all wells from each of the first five images (all wells from the first hour—during the growth lag phase). Including all wells within the first hour provides a wider array of illumination and camera angle conditions, improving negative control distributions. At later time points, automated growth detection was accomplished by quantifying the ratio of the “within group” variance of all of the negative controls (including all wells from the first hour of data) to the “between group” variance, which includes both positive control wells (H11, H12), all negative controls in each image, and all negative control wells from all prior time steps. A Fisher linear discriminant was calculated to maximize the distance between means of the growth and no-growth classes while minimizing the within-class variance. Growth-positive and growth-negative group labels for the Fisher discriminant were defined by the positive and negative control wells, respectively. If one assumes that positive and negative growth distributions are similar, one would expect a discriminant threshold of ~2 to distinguish (means are separated by approximately twice the variance). We found that a discriminant of 1.8 was sufficient for reliably separating growth-positive wells from growth negatives. Thus, the first time point beyond 2 h that yielded a discriminant value greater than or equal to 1.8 was a trigger to begin reporting MICs for that and all subsequent time points. This discriminant value, greater than or equal to 1.8, was used as the critical value to initiate MIC recording for all ChroMIC assays and was not used further in discriminating classes, as the SVM transitions into this discriminatory role for all test and control wells, labeling each well as growth-positive or growth-negative. For each frame after reporting (determining and displaying MICs in real time) has begun, all data were rotated into the same principal components space, and a grid search was performed to find the optimal SVM discriminant. SVM-derived probabilities of being in each class were used to assign growth (+) or no growth (−) for all test and control wells. This grid search enables the construction of an optimal SVM surface by iterating over a wide range of possible model hyperparameters and optimizing recall (maximizing true positives) to best separate positive (dark red) from negative (bright red) wells. Antibiotic concentrations increase from left to right along each row. Thus, at any given time point, the left-most well in each row that maintains bright red color (no growth) was taken as the MIC. Since each microtiter plate includes both positive and negative controls, color similarity to these controls is used to directly label wells as growth-positive or growth-negative.

### Bayesian updating and support vector machine probabilities

Over time, wells separate in color space due to spectral changes indicative of bacterial growth. Principal component overlap with paired positive and negative control wells classifies growth-positive wells with high accuracy, lessening bias in determining the growth class of intermediately colored wells. SVM probabilities were extracted from this initial model through mapping onto a sigmoid function across the discriminative boundary. As antibiotic concentration gradient increases along each row of the microtiter plate from left to right, the initial SVM well probabilities were used to update the classification of each well (growth-positive/growth-negative). For a given antibiotic, if a certain well is labeled growth-positive, this informs the probability that the well immediately to its right (2× higher concentration) is also positive, enabling Bayesian updating of growth-positive labels, as described more fully in the SI. The Bayesian-updated SVM probabilities were then calculated and used for final growth/well labels and MIC determinations, thereby reducing false positives due to the expected low frequency of growth at a high antibiotic concentration, without growth also being observed at lower antibiotic concentrations. In addition, this Bayesian update methodology accounts for well-skipping that may result due to bacterial growth at higher antibiotic concentrations, while skipping lower concentrations, and it self-adjusts to report reliable MICs by eliminating skipped wells.

### Gram stain and bacteria identification

Blood cultures from routine patient care were incubated using a BACT/ALERT continuously monitored blood culture instrument (BioMérieux Inc., Durham, NC) in the clinical microbiology laboratory at Emory University Hospital. Once flagged as positive, Gram stains were performed using standard dye staining and microscopic observation (30). Monomicrobial, aerobic, positive cultures showing Gram-negative rods from adult patients were used in the study if processed within 8 h of turning positive, under a residual clinical specimen protocol approved by the Emory University Institutional Review Board (IRB00093057). Only the first positive blood culture of any given patient was included in the study. In parallel with the ChroMIC experiments, cultures were plated, colonies were picked for mass spectrometry-based ID (VITEK MS, BioMérieux Inc., Durham, NC), and automated susceptibility testing was performed using VITEK 2 system with AST-GN74 card (BioMérieux Inc., Durham, NC), all according to our clinical laboratory standard operating procedures. As our study aimed to determine susceptibility directly from positive blood culture and compare ChroMIC accuracies to those from VITEK 2, polymicrobial samples were only identified after being run. However, susceptibilities could not be determined for such samples without plating and colony-picking–based separation. Thus, while we obtained patterns suggesting successful AST tests, these 4.6% of samples (4/87) were excluded from the study as they were not analyzable by VITEK 2 without separation. Typically, polymicrobial blood cultures account for less than 10% of BSIs (31), and additional methods are needed for ASTs on such samples to be considered accurate and reliable.

### Bacterial isolation and CFU/mL estimation

We tested our assumption that blood cultures typically flag positive close to ~5 × 10^8^ CFU/mL (32) by performing all ChroMIC assays with 1,000-fold dilution directly from positive cultures. Actual CFU/mL in positive blood cultures, and therefore actual bacterial concentrations used for assays, were retrospectively determined by counting colonies from plating serially diluted positive blood cultures on LB agar (Lennox; Sigma-Aldrich, St. Louis, MO) and incubating overnight at 37°C (Fig. S2). Bacterial colonies recovered from this plating step were used for BMD-based MICs.

**Fig 2:**
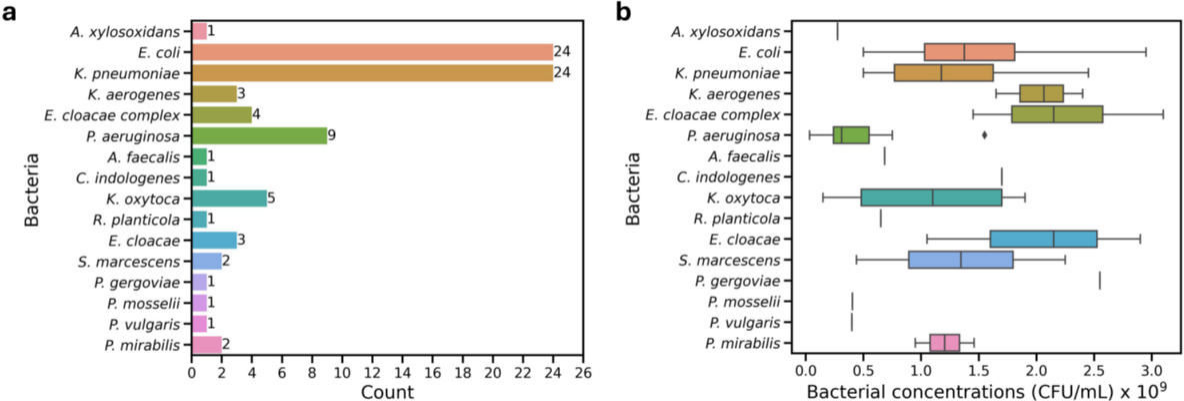
Pathogens in analyzed positive blood cultures (83 samples). (**a**) Distribution of Gram-negative bacteria causing BSIs in obtained blood cultures. (**b**) Boxplot showing bacterial concentration (CFU/mL) in the positive blood culture of each type of Gram-negative bacteria.

### BMD of bacteria isolated from positive blood cultures

BMDs were performed according to CLSI guidelines (27). Briefly, a single bacterial colony was inoculated in CAMHB medium and incubated at 37°C and ~225 rpm for ~3 h in a MaxQ 4000 incubator shaker (Thermo Fisher Scientific, Waltham, MA). After incubation, the sample was diluted in CAMHB, and the optical density at 600 nm was adjusted to ~0.002 (Shimadzu, UV-2401 PC, UV-VIS Recording Spectrophotometer) which corresponds to ~10^6^ CFU/mL. Then, 100 µL was dispensed in each well of a 96-well plate containing serially diluted antibiotics, yielding a final volume of 200 µL per well. MICs were determined visually at 18 h, in accordance with CLSI guidelines (27).

### Data analysis

Images were taken once every 15 minutes over an 18-hour period. Categorical and essential MIC agreements (CA and EA, respectively) (33) were calculated for ChroMIC MICs vs. those from BMD for each antibiotic at each time point. Importantly, because BMD and VITEK 2 only give a final result, our faster results were compared against these standard long-term results at every measured time point to assess both categorical and essential agreements. Because ChroMIC measured a much wider concentration range, we imposed the much narrower VITEK 2 concentration ranges on our MICs for essential agreement (EA) determinations.

ChroMIC and VITEK 2 error rates were calculated using the gold standard BMD with minor errors (mE), major errors (ME), and very major errors (VME) defined according to FDA guidelines (34). The CLSI breakpoints were used to determine if the given drug-bacteria combination falls into susceptible, intermediate, or resistant categories (35).

### Potential clinical impact assessment

Culture result timestamps and antimicrobial administration records were retrospectively retrieved from the medical record via a clinical data warehouse, according to protocols approved by the Emory University Institutional Review Board (IRB00093057, STUDY00006467, and STUDY00007793). Since a bacteremia episode often involves more than one positive blood culture specimen, we used the timestamp for the first reported blood culture Gram stain result in the episode. This occurs following notification of a positive blood culture by the BacT/ALERT system, retrieval of the specimen, and execution of the Gram stain procedure, thereby representing the approximate time of the first positive culture in the bacteremia episode. To characterize the delay from the first positive blood culture to AST-informed adjustment of antimicrobial therapy, the antimicrobial record and AST results were reviewed by an infectious disease physician, and therapeutic changes were classified as “escalation,” “de-escalation,” or “no change.” Cases were excluded from this analysis if there were other culture data to guide therapy (e.g., concurrent treatment for another organism with more extensive antimicrobial resistance); if the patient’s death, discharge to hospice, or discharge against medical advice precluded changes to antimicrobial therapy; if AST revealed an opportunity to de-escalate therapy but the managing team did not; or if the patient was not admitted to the hospital for treatment of the bloodstream infection. The time of administration of the first dose of the appropriate antimicrobial agent and the time of AST report were identified, and the “information delay” time was calculated from the Gram stain report to first dose of appropriate therapy or reporting of AST results (whichever occurred first). Hospital progress notes were reviewed if needed to clarify the managing team’s clinical reasoning.

## RESULTS AND DISCUSSION

### Estimation of bacterial concentration

For ASTs, the CLSI standard bacterial density should be ~5 × 10^5^ CFU/mL (27). Validating our approach of applying a blanket 1,000-fold dilution to positive blood cultures for performing ChroMIC assays, these cultures exhibited relatively consistent bacterial densities (Fig. 2b), with both the mean and median equal to 1.3 × 10^9^ CFU/mL and a standard deviation of 0.7 × 10^9^ CFU/mL. Thus, our blind dilution typically resulted in ~1 × 10^6^ CFU/mL in each well.

### MIC determination using ChroMIC assays

**Fig 3:**
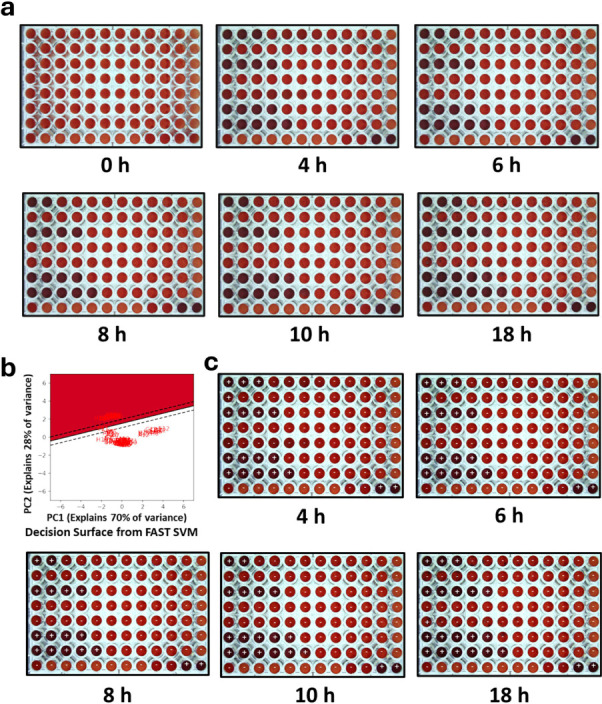
Automated real-time ChroMIC assay of a blood culture containing *E. coli*. (**a**) ChroMIC images at different times (0 h, 4 h, 6 h, 8 h, 10 h, and 18 h). (**b**) FAST SVM generated the Bayesian-updated decision surface and the output MIC for each time point. (**c**) Bayesian-updated predicted labels by FAST SVM. + sign indicates bacterial growth, and – sign indicates no bacterial growth at the given antibiotic concentration in that well.

Commercial blood culture bottles typically indicate microbial growth by directly or indirectly detecting gas production that occurs as a result of metabolic activity. Once sufficient growth-induced respiration occurs, bottles are flagged positive. We (36) and others (28, 29) noticed that positive blood cultures darken in color as the oxy-/deoxy-hemoglobin ratio is altered, due to bacterial respiration consuming O_2_ and producing CO_2_. These dissolved gases convert Hb-O_2_ to Hb with a corresponding change from bright to dark red. Successively bubbling CO_2_ then O_2_ through a blood culture containing blood products shows the reversibility of this spectral change (Fig. S1). Initially shown to indicate high levels of bacteria or presence in blood or blood cultures (37, 38), we utilize this bacterial respiration-induced spectral change as the indicator in our ASTs directly from diluted Gram-negative aerobic positive blood cultures. The 1,000-fold dilution required to obtain the appropriate bacterial amount makes the color change unobservable in diluted blood culture. However, dilution in media with blood added as contrast uniquely enables bacterial growth in the presence of antibiotics to be directly assayed and MICs to be directly determined colorimetrically. Typical growth-based MIC determinations from the SVM discriminant are shown in Fig. 3 with Bayesian-updated (+ or −) labels on each well (details in Methods). Extracted MICs for this sample are given in Table 1.

**TABLE 1:** Real-time Bayesian-updated MICs at multiple time points of the corresponding images in Fig. 3a along with BMD and VITEK 2 results

Antibiotics	MIC (4 h) (µg/mL)	MIC (6 h) (µg/mL)	MIC (8 h) (µg/mL)	MIC (10 h) (µg/mL)	MIC (12 h) (µg/mL)	MIC (18 h) (µg/mL)	BMD (µg/mL)	Vitek 2 (µg/mL)
Ceftazidime	0.25	0.25	0.125	0.125	0.125	0.25	0.125	≤1
Meropenem	0.0625	≤0.03125	≤0.03125	≤0.03125	≤0.03125	≤0.03125	≤0.03125	≤0.25
Tobramycin	0.5	0.5	0.5	0.5	1	1	1	≤1
Levofloxacin	≤0.03125	≤0.03125	≤0.03125	≤0.03125	≤0.03125	≤0.03125	<=0.03125	≤0.125
Cefepime	≤0.03125	≤0.03125	≤0.03125	≤0.03125	≤0.03125	0.0625	0.0625	≤1
Gentamicin	0.5	0.5	0.5	1	1	1	1	≤1
Amikacin	1	1	1	2	2	2	2	≤2

### ChroMIC accuracy

**Fig 4:**
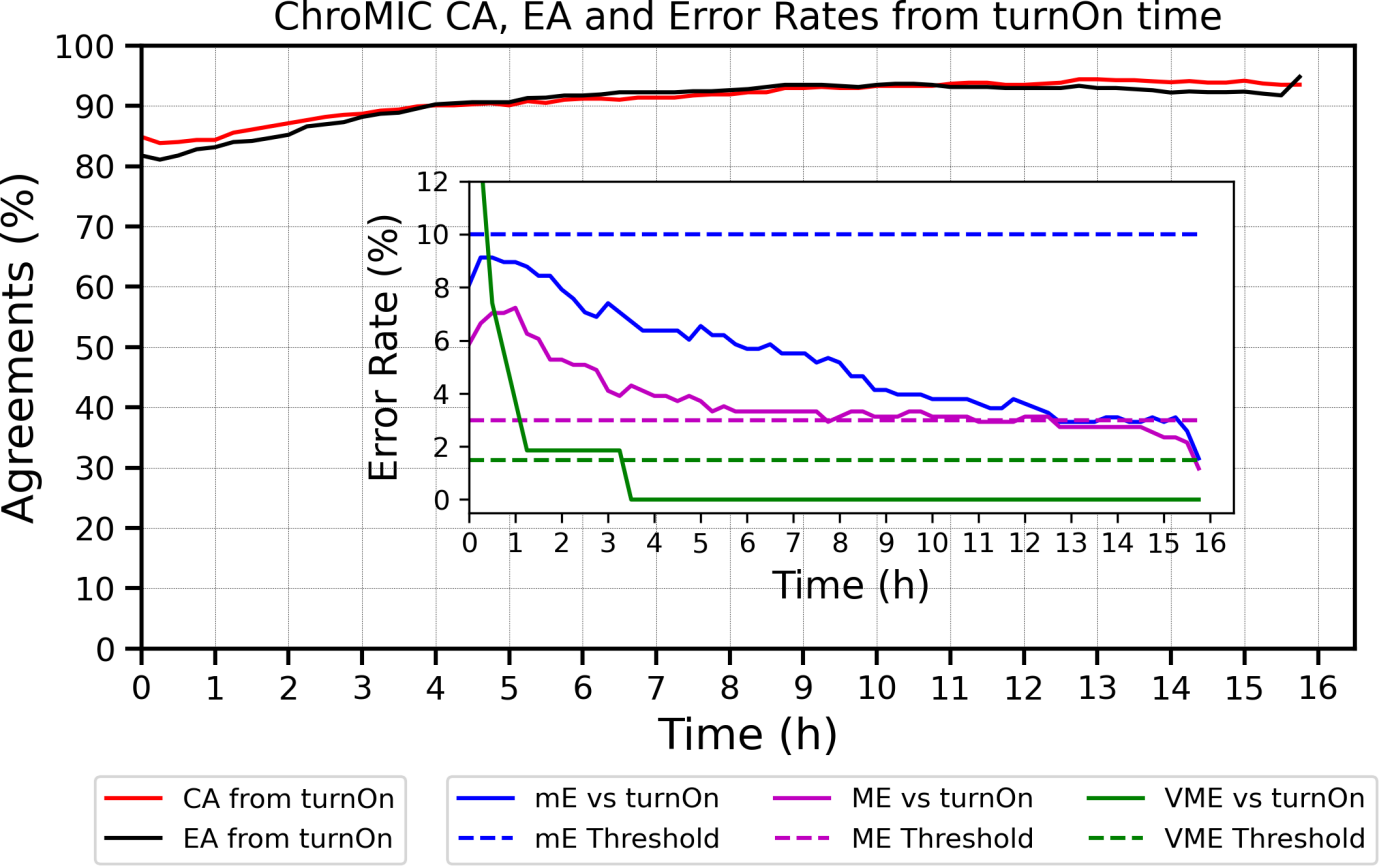
Essential and categorical agreements vs. BMD as a function of time from growth first being registered (turnOn) in positive control wells. Both EA and CA exceed 90% within 4 h of initial growth in positive wells. The inset shows properly normalized minor, major, and very major error rates from growth initiation across all 83 samples with seven antibiotics each. Minor errors are normalized by all bacteria–antibiotic pairs. Major errors are normalized by BMD-susceptible strains, and VMEs are normalized by BMD-resistant strains. Errors vs. start of experiment are shown in Fig. 5.

**Fig 5:**
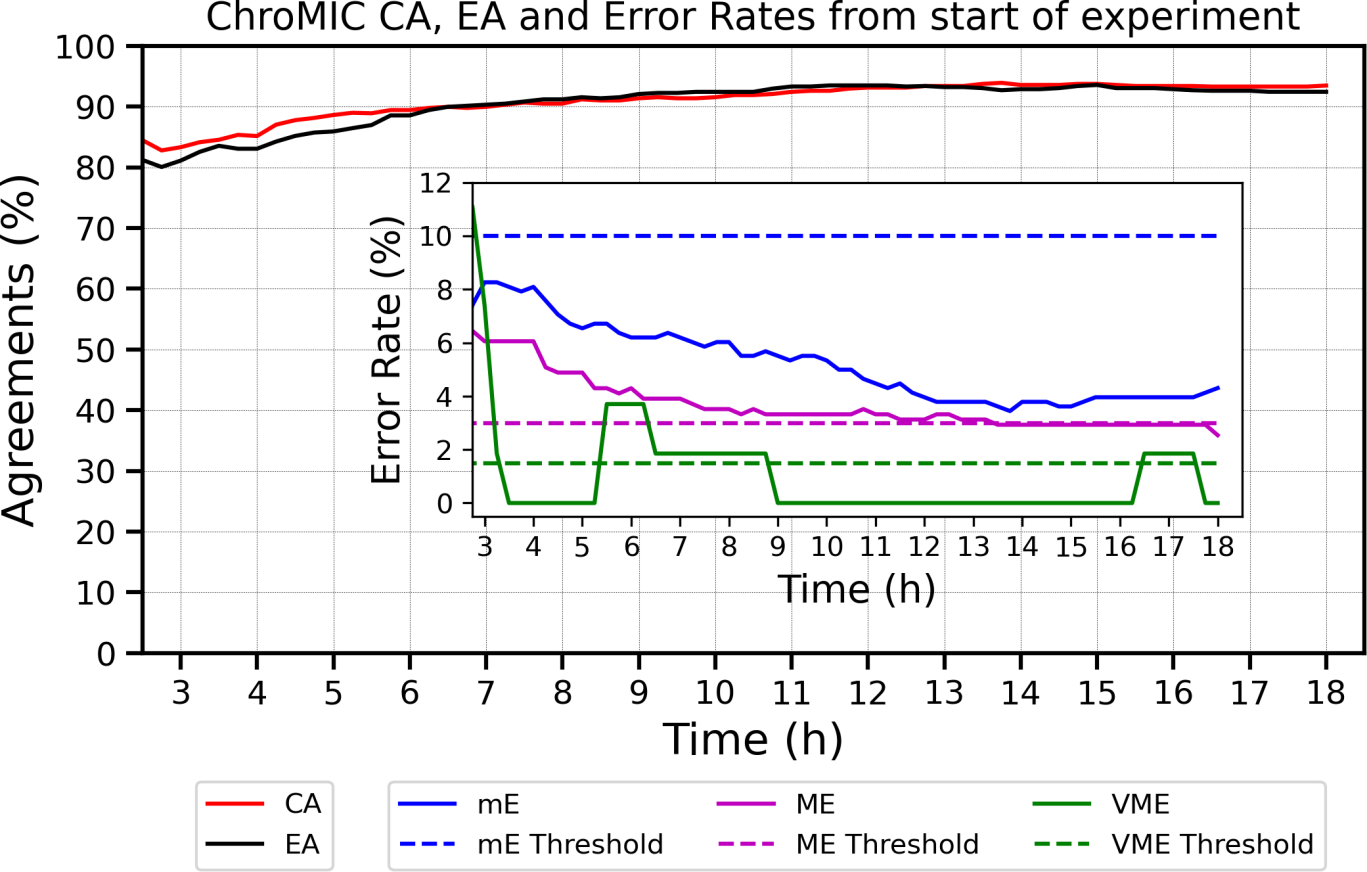
Evaluation of ChroMIC results with standard BMD from start of experiment. ChroMIC CA (solid red curve), EA (solid black curve), and error rates (inset) are shown with respect to BMD. The dashed blue, magenta, and green lines represent threshold errors of 10%, 3%, and 1.5% for mE, ME, and VME, respectively, and the solid color curves are the corresponding ChroMIC errors.

ChroMIC results on Gram-negative rods (83 samples total) were obtained directly from freshly turned (within 8 h) blood cultures without prior knowledge of bacterial ID, MICs, or bacterial densities in the obtained positive cultures. ChroMIC MICs were blindly compared against the BMD results. Also, VITEK 2 MICs used for treatment guidance at Emory University Hospitals were compared against BMD. BMD-determined MICs from pure colonies were obtained after ChroMIC and VITEK 2 results. Using BMD as the gold standard, ChroMIC EA was calculated across the VITEK 2 ranges for each antibiotic. For example, if ChroMIC reports 0.125 µg/mL for amikacin, we would adjust this to the VITEK 2 range of ≤ 2 µg/mL and gage whether BMD is within a factor of 2 of this adjusted ChroMIC value. Using this approach, EA with BMD exceeds 90% within 4 h of registering growth in the positive control wells (Fig. 4) and after 7 h from the start of susceptibility determination (Fig. 5). BMD, however, is a much longer-time single-point measurement of susceptibility, performed after overnight plating followed by 18–24 h of antibiotic-challenged growth.

**Fig 6:**
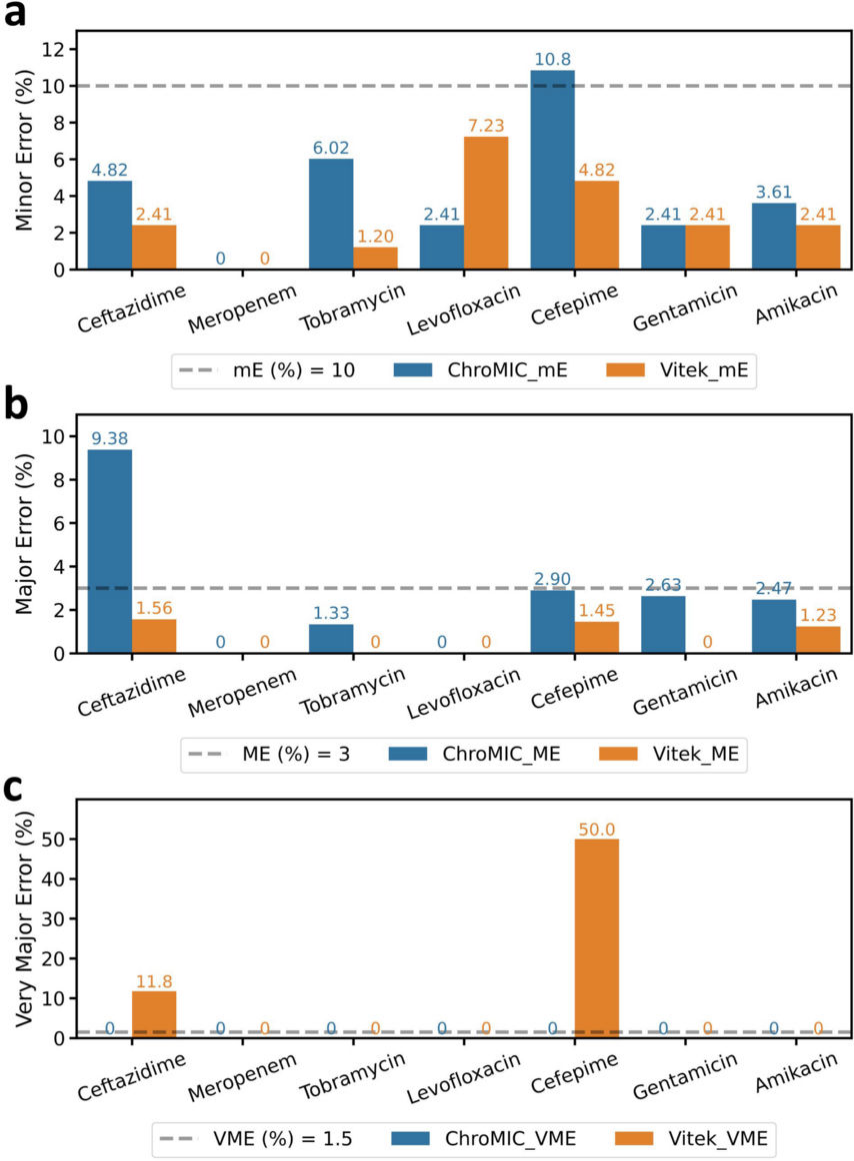
Breakdown of ChroMIC and VITEK 2 endpoint error rates as a function of antibiotics used relative to standard BMD. Blue bar indicates ChroMIC error rates at 18 h after blood culture positivity, and orange bar indicates VITEK 2 error rates obtained after ~18 h plating/growth followed by 6–18 h of susceptibility determination, >24 h after blood culture positivity. (**a**, **b **and **–c**) ChroMIC (blue) and VITEK 2 (orange), minor (mE), major (ME), and very major (VME) errors for each antibiotic (the inset dashed line indicates FDA threshold for each error type).

ChroMIC categorical agreement with BMD shows >90% accuracy after 4 h from growth being observed in positive controls (Fig. 4) and after 7 h from the start of the assay (Fig. 5), with very low minor, major, and very major errors (Fig. 4). Plotting errors from the time growth is first observed for each strain clearly shows that errors are higher soon after growth begins, while errors at longer times from initiation of the AST assay typically appear from slower-growing organisms. Coupled with our automated growth detection, aligning all strains based on time from growth detection in positive control wells (and hence MICs being recorded, which we labeled as “turnOn” in Fig. 4) provides a better indication of true error rates and how long one must wait for accurate results. After 3 h from growth being observed, only the major errors exceeded the FDA-approved thresholds (34), but these also decreased at longer times. Of the seven tested antibiotics, ceftazidime is the main contributor to the high ME rate (Fig. 6b), with bacteria occasionally showing early growth at high ceftazidime concentrations but exhibiting much lower MICs at the final 18-hour time point. Although ceftazidime better matches with BMD at longer times, calculating CA without ceftazidime significantly decreases the ME rate well below FDA thresholds (Fig. S4a and S4b). Importantly, no VMEs are observed after 3.5 h of growth first being registered in the AST for ChroMIC (Fig. 4 Inset), yet VITEK 2 produces several of these potentially catastrophic VMEs (Fig. 6c and Table S2). For comparison, VITEK 2 EA and CA (using BMD as the standard) were 95.4% (Table S2) when performed after ~18 h of subculturing and 8–16 h AST, resulting in a delay of >24 h relative to ChroMIC. VITEK 2, of course, only provides a single end-point result (Table S2). At this long time point, VITEK 2 minor and major errors are below the recommended thresholds (34) for all seven antibiotics (Fig. 6a and b, respectively). Using ChroMIC, only cefepime mE (10.8%, Fig. 6a) and ceftazidime ME exceeded the recommended thresholds (9.38%, Fig. 6b). Although VITEK 2 does not exhibit any VME for amikacin, gentamicin, levofloxacin, tobramycin, and meropenem, it greatly exceeds the VME threshold for both cefepime (50%) and ceftazidime (11.8%) (Fig. 6c). These high VMEs of VITEK 2 for ceftazidime and cefepime are consistent with findings from several other studies on Gram-negative bacteria (14, 39, 40). These false susceptibilities (VMEs) are completely avoided using ChroMIC (Fig. 6c), while ChroMIC simultaneously yields MICs in a much shorter time with high overall accuracies.

**Fig 7:**
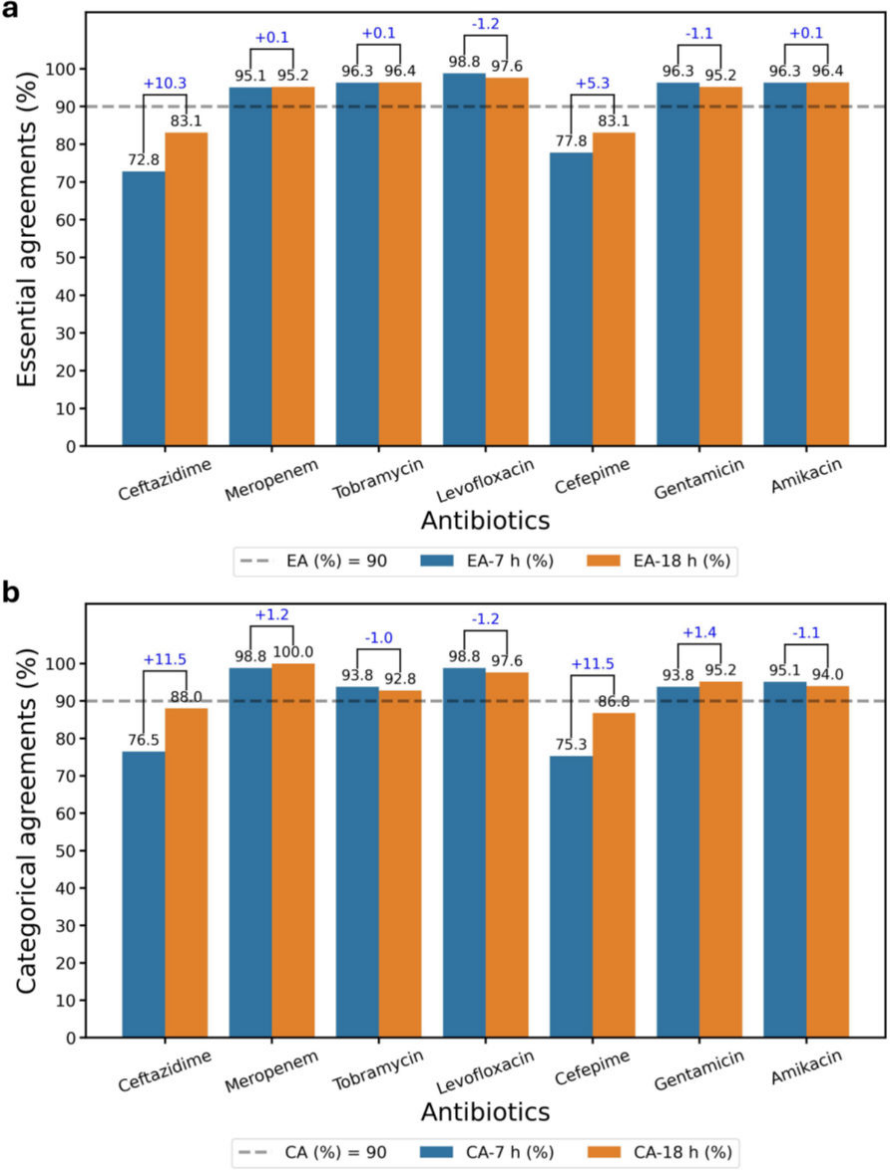
ChroMIC EA and CA as a function of antibiotic used at 7 h and 18 h after blood culture positivity. Breakdown of ChroMIC (**a**) EA at 7 h (blue bar) and 18 h (orange bar) and (**b**) CA at 7 h (blue bar) and 18 h (orange bar) with respect to BMD. The numbers at the top of paired bars are the difference in EA or CA, with the positive and negative sign indicating increase and decrease of agreements at 18 h with respect to 7 h, respectively. The dashed lines indicate 90% threshold.

**Fig 8:**
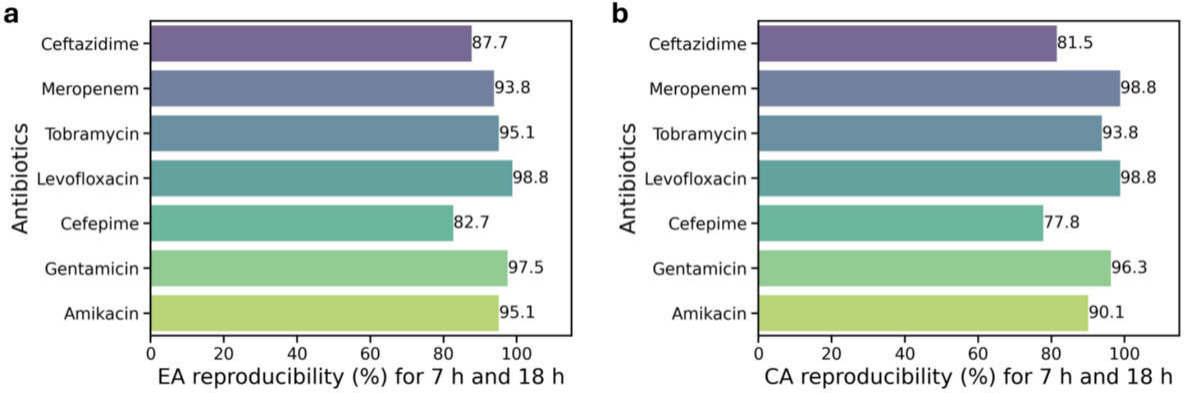
ChroMIC (**a**) EA and (**b**) CA reproducibility percentage between 7 h and 18 h for the samples that recorded MICs within 7 h from the start of assay (Fig. S3).

Out of 83 samples studied, 81 samples grew sufficiently rapidly such that MIC reporting began within 6 h from the start of assay. Two samples (one *P. aeruginosa* and one *P. mirabilis*) only began reporting MICs starting at 12.75 h and 16.5 h, respectively (Fig. S3). Even though VMEs appear high from the start of the experiment, normalizing to the start of growth (turn-on time) shows that all VMEs disappear very quickly after growth begins for each sample, with no VMEs being observed after 3.5 h from turn-on time (Fig. 4, Inset). Reporting accuracies and errors from the time when positive growth wells show growth normalizes for these differences. While 7 h from the start of the AST yields excellent overall accuracies exceeding 90% for EA and CA, cefepime and ceftazidime accuracies continue to improve from 7- to 18-hour time points (Fig. 7a and b, Table 2). For all other antibiotics, ChroMIC EA and CA at 7 h from the start of the assay exceeded 90% and remained largely constant through the 18-hour time point (Fig. 7a and b). A comparison of 7- to 18-hour MIC reproducibility for each antibiotic is shown in Fig. 8, suggesting that ChroMIC can yield highly accurate MICs with a much shorter time to results.

**TABLE 2:** ChroMIC agreements and error rates at 7 h and 18 h directly from positive blood culture

Antimicrobial	Time (h)	EA (%)	CA (%)	mE (%)	ME (%)	VME (%)
Ceftazidime	7	59/81 (72.8)	62/81 (76.5)	9/81 (11.1)	10/62 (16.1)	0/17 (0)
	18	69/83 (83.1)	73/83 (88.0)	4/83 (4.82)	6/64 (9.38)	0/17 (0)
Meropenem	7	77/81 (95.1)	80/81 (98.8)	1/81 (1.23)	0/77 (0)	0/3 (0)
	18	79/83 (95.2)	83/83 (100)	0/83 (0)	0/79 (0)	0/3 (0)
Tobramycin	7	78/81 (96.3)	76/81 (93.8)	3/81 (3.70)	1/73 (1.37)	1/6 (16.7)
	18	80/83 (96.4)	77/83 (92.8)	5/83 (6.02)	1/75 (1.33)	0/6 (0)
Levofloxacin	7	80/81 (98.8)	80/81 (98.8)	1/81 (1.23)	0/67 (0)	0/11 (0)
	18	81/83 (97.6)	81/83 (97.6)	2/83 (2.41)	0/68 (0)	0/12 (0)
Cefepime	7	63/81 (77.8)	61/81 (75.3)	15/81 (18.5)	5/67 (7.46)	0/10 (0)
	18	69/83 (83.1)	72/83 (86.8)	9/83 (10.8)	2/69 (2.90)	0/10 (0)
Gentamicin	7	78/81 (96.3)	76/81 (93.8)	3/81 (3.70)	2/74 (2.70)	0/6 (0)
	18	79/83 (95.2)	79/83 (95.2)	2/83 (2.41)	2/76 (2.63)	0/6 (0)
Amikacin	7	78/81 (96.3)	77/81 (95.1)	3/81 (3.70)	1/79 (1.27)	na^*a*^
	18	80/83 (96.4)	78/83 (94.0)	3/83 (3.61)	2/81 (2.47)	na^*a*^
Overall	7	513/567 (90.5)	512/567 (90.3)	35/567 (6.17)	19/499 (3.81)	1/53 (1.89)
	18	537/581 (92.4)	543/581 (93.5)	25/581 (4.30)	13/512 (2.54)	0/54 (0)

^*a*^ No resistant strains were observed for amikacin for these samples.

For all 83 patient samples studied, the overall EA, CA, mE, and ME of both ChroMIC and VITEK 2 were all within FDA thresholds (Table 2), but VITEK 2 ASTs had to be performed after an additional ~18 h of subculturing post-positive blood culture. Crucially, however, VITEK 2 exhibited a very high VME of 13.0% (Table S2) on these same samples—a value that greatly exceeds the FDA-recommended threshold of 1.5% (34). In comparison, ChroMIC produced zero VMEs on these same samples, suggesting that it may have significant advantages when compared to or used in conjunction with current methods.

### Potential impact on patient treatment

**Fig 9:**
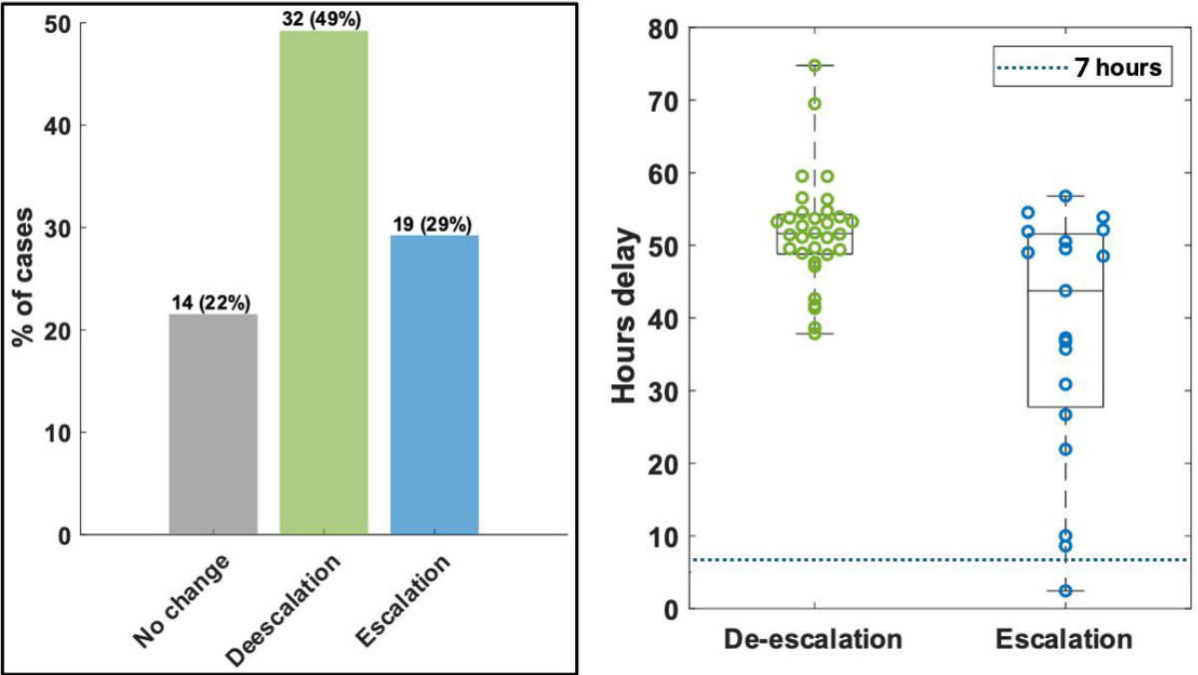
(Left) Cases corresponding to patient cultures studied where treatment was changed. (Right) Time after blood culture positivity at which de-escalation and escalation were initiated vs. when ChroMIC would have enabled treatment revision (dotted line at 7 h after blood culture positivity).

Sixty-five (78.3%) of 83 cultures were included in an impact analysis (Tables S3 through S9) of how faster susceptibility results might have affected patient care. Therapeutic changes were classified as escalation in 19 (29.2%) and de-escalation in 32 (49.2%) (Fig. 9). The median time to escalation from Gram stain result was 43.8 h (IQR 27.7–51.6), and 18 (93.3%) of 19 escalation events occurred more than 7 h after the Gram stain result. The median time to de-escalation was 51.6 h (IQR 48.8–54.2), with 100% of 32 de-escalation events occurring more than 7 h after the Gram stain result. Thus, almost every therapeutic change made in this sampling of bloodstream infections could have been made sooner with the use of the ChroMIC assay, where results are expected within 7 h. This earlier de-escalation helps limit the use of broad-spectrum agents, an important principle in antimicrobial stewardship, while earlier escalation, in many cases, could be lifesaving. While it is likely that faster susceptibility could improve patient care and lower hospital costs, further studies will be needed to assess the true impact of the ChroMIC system.

### Conclusion

Phenotypic ASTs remain the gold standard for determining the susceptibility of bacterial bloodstream pathogens. The urgency of rapid treatment, coupled with long AST turnaround times (30 h or greater even with automated systems) from positive blood culture, leads to often untargeted treatment and increased sepsis-induced morbidity and mortality. In contrast, we have developed a simple colorimetric assay directly from positive blood culture that yields accurate MICs within 7 h of blood culture positivity. By removing the need for plating and using the oxy/deoxy hemoglobin color change, we obtain highly accurate MICs in approximately one-fifth the time of automated systems typically used in clinical microbiology labs. These results on Gram-negative species indicate that ChroMIC is a promising technology for rapid, direct-from-positive-blood-culture ASTs. With future studies across a wider range of Gram-negative species and extension to Gram-positives and fungi, ChroMIC’s capabilities can be further elucidated. Although ChroMIC yields automated real-time MICs from the start of each experiment with pre-prepared substrates, incorporation into clinical workflows will require automated sample handling before it can be conveniently implemented in regular clinical settings. Correlating ChroMIC speed improvements with treatment decisions impacted by traditional ~30+ hour susceptibility results, a surprisingly large fraction of patients would have been likely to significantly benefit from the faster ChroMIC susceptibility results.

## Data Availability

All MIC data (BMD, VITEK 2, and ChroMIC) for each sample isare available on request. ChroMIC MIC data vs. time for all 83 samples are available at https://sites.gatech.edu/dicksonlab/supplementary-data-for-publications/. Software used for MIC determination was written specifically for determining MICs from the acquired images and is described and detailed in the Materials and Methods and Supplemental material of this article.

## SUPPLEMENTAL MATERIAL

Supplemental material spectrum.02147-25-s0001.pdf Supplemental methods; Fig. S1 to S4; Tables S1 to S9.

ASM does not own the copyrights to Supplemental Material that may be linked to, or accessed through, an article. The authors have granted ASM a non-exclusive, world-wide license to publish the Supplemental Material files. Please contact the corresponding author directly for reuse.
