# Supplementary material for: Rapid colorimetric antimicrobial susceptibilities direct from positive blood culture for Gram-negative bacteria
Source: Microbiol Spectr. 2025 Sep 19;13(11):e02147-25. doi: 10.1128/spectrum.02147-25 (PMC12584658; doi:10.1128/spectrum.02147-25)
Supplement: Supplemental material — Supplemental methods; Fig. S1 to S4; Tables S1 to S9. [file spectrum.02147-25-s0001.pdf]

Supplementary Information for:

Rapid colorimetric antimicrobial susceptibilities direct from positive blood culture for Gram-negative bacteria

Prakash C. Khanal[†], Joseph C. Richardson[†], Katherine G. Richardson[†], Greg L. Damhorst[^], Owen J. Oertell[†], Alexandra Filbrun[†], Eileen M. Burd[‡], and Robert M. Dickson*[,†]

[†]School of Chemistry & Biochemistry and Institute for Bioengineering and Bioscience, Georgia Institute of Technology, ^Division of Infectious Diseases, Department of Medicine, Emory University School of Medicine, and [‡]Department of Pathology and Laboratory Medicine, Emory University School of Medicine, Atlanta, GA, USA

*Email: dickson@chemistry.gatech.edu

Supplemental methods:

Bayesian updating of growth labels.

If a well shows growth, it is likely that the lower antibiotic concentration well to its left should also be growth positive. Thus, we use conditional probabilities (Bayesian statistics) to update growth predictions. Bayes' theorem allows calculation of the probability of growth in a higher concentration well, given that there is or is not growth in a lower antibiotic concentration well. Thus, the probability that B is true given that A is true (P(B|A)) is the probability of A being true given that B is true (P(A|B)), multiplied by the probability that B occurs (P(B)), and normalized by the probability of A occurring (P(A)): $P(B|A) = \frac{P(A|B)P(B)}{P(A)}$. In this situation, P(B) is the prior probability, P(B|A) is the posterior (or updated) probability, given the prior information. Using a sigmoid function across the SVM boundary, prior probabilities of each well being growth positive or growth negative are determined by distance from the optimal SVM boundary. The posterior probability for each well (the updated quantity we want to predict) is computed by taking the product of the prior probability (as predicted by our SVM model) and the likelihood (the probability of A and B happening, normalized by the probability of A happening). This likelihood computation comes from considering the experimental frequency of each of the four possible combinations of positive and negative labels, PP, PN, NP, NN (with P being the examples that were labeled positive from the SVM model and N being the examples that were labeled negative from the model) along this concentration gradient. The relative abundance of each of these possible pairs informs how likely it is that "*given that well i-1 was determined to be positive, what is the probability that well i is also positive*".

The likelihood computation here is performed along the increasing concentration gradient for every antibiotic, so the relative abundance of each of the four possible combinations is enumerated per plate, at every time point. In this schema, the SVM probabilities are updated with this Bayesian methodology to reinforce the physical and biological constraint that positive wells, at high antibiotic concentrations, should have positive examples preceding them, along the concentration gradient. This method allows false positive wells to be 'knocked down' due to the expected low frequency of having a positive example

at a high antibiotic concentration, with previous examples being predicted as negative examples at lower antibiotic concentrations.

Antibiotics used and plate layout for ChroMIC and BMD assays.

Table S1. Antibiotics used for ChroMIC assays.

Antibiotic	Drug class	Manufacturer
Ceftazidime	Cephalosporin	RPI corp., Mount Prospect, IL
Meropenem	Carbapenem	Tokyo Chemical Industry, Tokyo, Japan
Tobramycin	Aminoglycoside	RPI corp., Mount Prospect, IL
Levofloxacin	Fluroquinolone	Alfa Aesar, Haverhill, MA
Cefepime	Cephalosporin	Chem-Impex Int'l, Wood Dale, IL
Gentamicin	Aminoglycoside	MP Biomedicals, Solon, OH
Amikacin	Aminoglycoside	MP Biomedicals, Solon, OH

Color change upon carbon dioxide production in growth medium, using human blood as contrast agent.

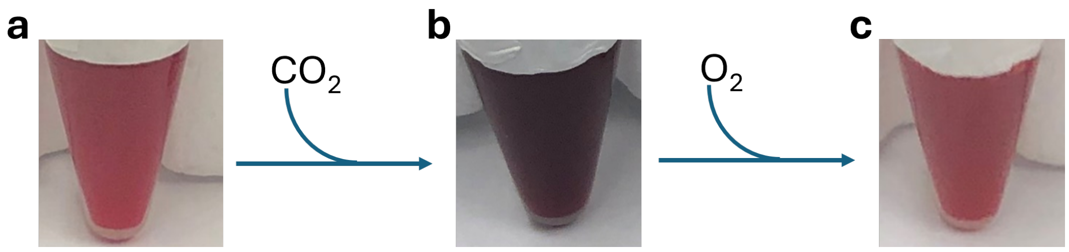

Figure S1. Color change of blood in CAMHB media purged with CO_2, followed by O_2 purging. **a.** Whole blood in CAMHB showing initial bright red color. **b.** Purging with CO_2 causes color change to dark red. **c.** Subsequent purging with O_2 turns the color back to bright red.

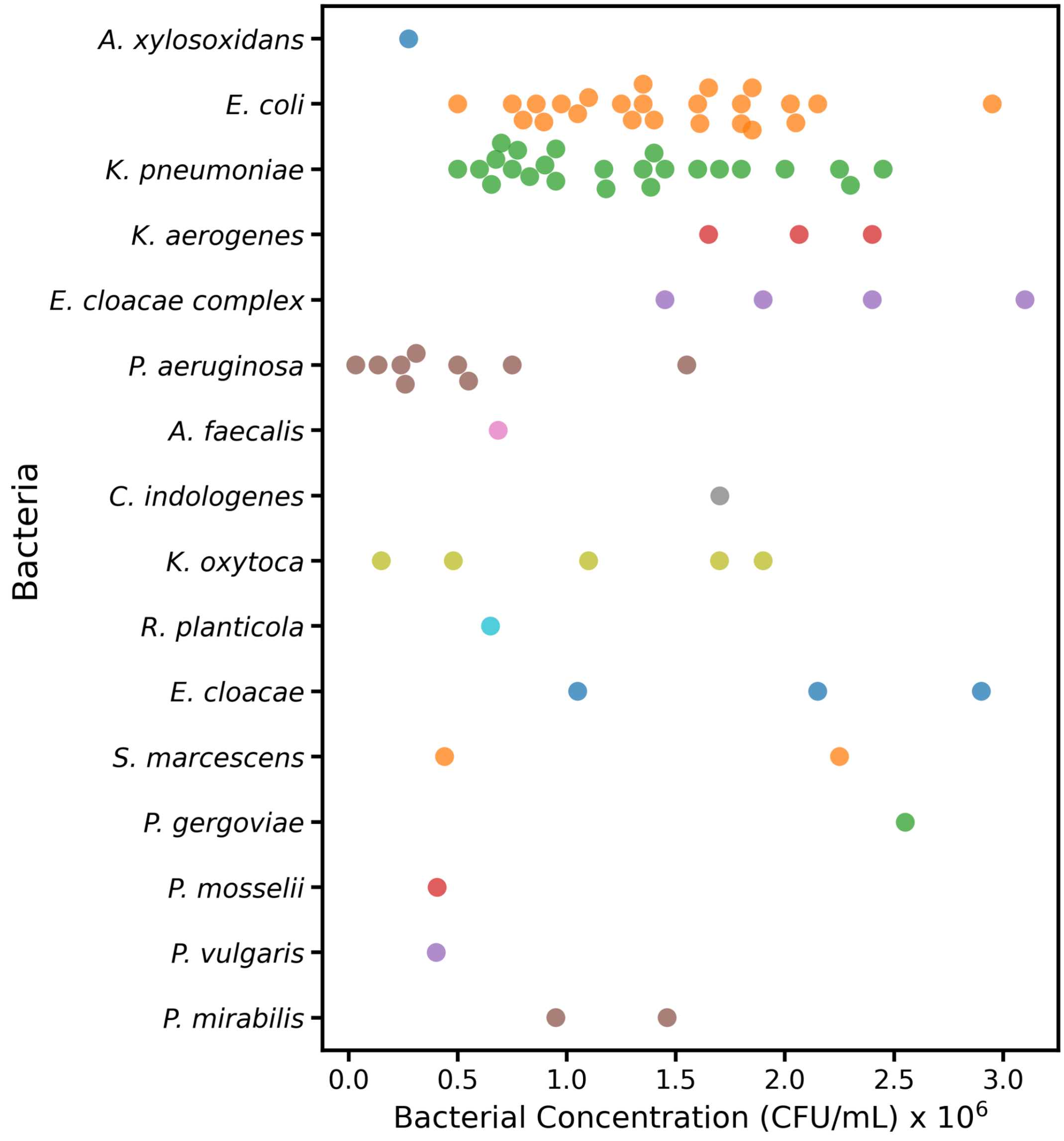

Figure S2. Estimation of bacterial concentrations (CFU/mL) at the start of ChroMIC assays for each of the studied 83 samples.

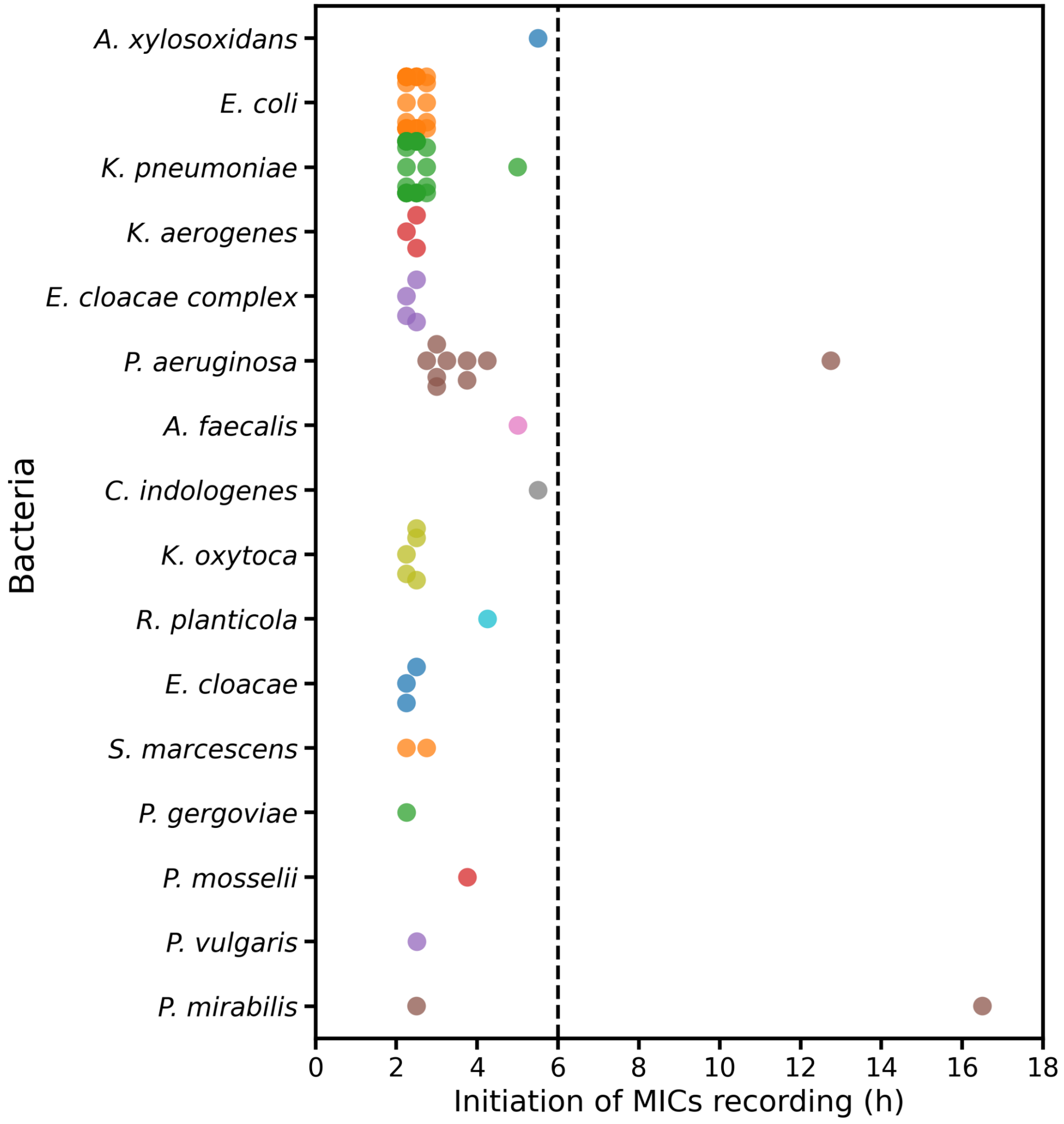

Figure S3. Initiation of ChroMIC MICs recording for the studied 83 samples. The left and right side of the dashed line indicates the initiation of MICs recording before (81 samples) and after 6 h (2 samples) from the start of assay.

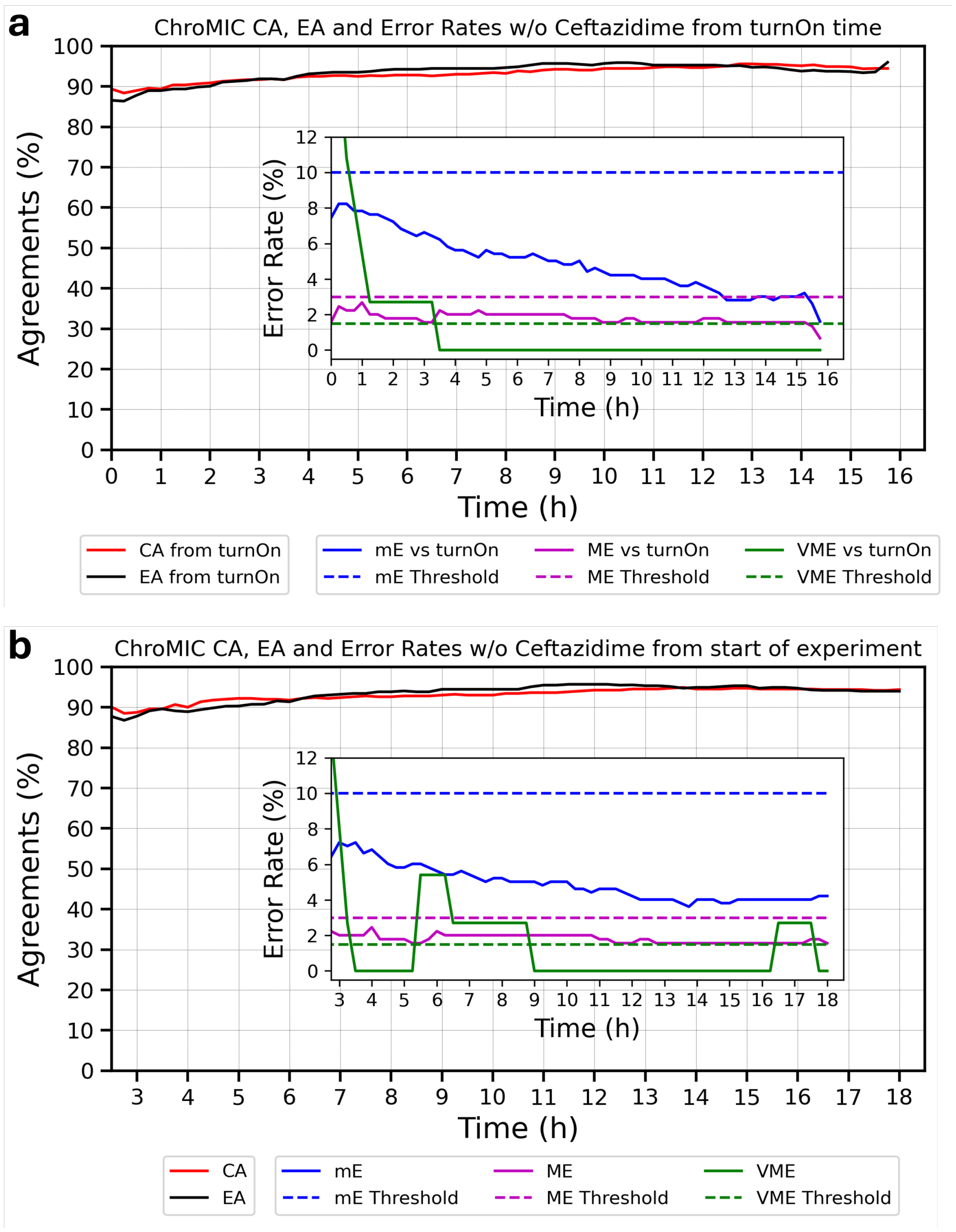

Figure S4. a Essential and categorical agreements vs. BMD as a function of time from growth first being registered (turnOn) in positive control wells without ceftazidime. The inset shows mE, ME, and VME from growth initiation for 83 samples and 6 antibiotics. **b** Essential and categorical agreements vs. BMD as a function of time from the start of experiment without ceftazidime. The inset shows the mE, ME and VME from the start of experiments for 83 samples and 6 antibiotics.

VITEK® 2 Essential agreement, Categorical agreement, and error rates as function of antibiotic used (after ~18-h plating/growth and 6-12-h susceptibility determination), >24 h after blood culture positivity, the numerator and denominator for each metric is in accordance with FDA definitions.[1]

Table S2. VITEK® 2 agreements and errors rates susceptibility results performed after ~ 18 h subculturing post-positive blood culture.

Antimicrobial	EA(%)	CA(%)	mE(%)	ME(%)	VME(%)
**Ceftazidime**	74/83 (89.2)	78/83 (94.0)	2/83 (2.41)	1/64 (1.56)	2/17 (11.8)
**Meropenem**	81/83 (97.6)	83/83 (100)	0/83 (0)	0/79 (0)	0/3 (0)
**Tobramycin**	83/83 (100)	82/83 (98.8)	1/83 (1.20)	0/75 (0)	0/6 (0)
**Levofloxacin**	80/83 (96.4)	77/83 (92.8)	6/83 (7.23)	0/68 (0)	0/12 (0)
**Cefepime**	73/83 (88.0)	73/83 (88.0)	4/83 (4.82)	1/69 (1.45)	5/10 (50.0)
**Gentamicin**	82/83 (98.8)	81/83 (97.6)	2/83 (2.41)	0/76 (0)	0/6 (0)
**Amikacin**	81/83 (97.6)	80/83 (96.4)	2/83 (2.41)	1/81 (1.23)	na*
**Overall**	554/581 (95.4)	554/581 (95.4)	17/581 (2.93)	3/512 (0.59)	7/54 (13.0)

* No resistant strains were observed for amikacin for the studied 83 samples.

Potential clinical impact of ChroMIC-determined susceptibilities

Therapeutic changes are indicated in Tables S3-S9 below, and were classified as “escalation,” “de-escalation,” or “no change.” Escalation included changes from an agent with a more limited range of antimicrobial activity to an agent with a broader range of antimicrobial activity, such as a switch to a later generation cephalosporin from an earlier generation cephalosporin and a switch to a carbapenem from any beta-lactam with beta-lactamase inhibitor (BL/BLI) or cephalosporin. Therapeutic changes from an agent to which the cultured bacteria was non-susceptible (as determined by VITEK® 2) to an agent with demonstrated susceptibility was also classified as escalation. A switch from piperacillin-tazobactam (PTZ) to cefepime was considered escalation if for the treatment of an organism with a high likelihood of harboring an inducible-AmpC beta-lactamase (even if PTZ was reported as susceptible), since this therapeutic change is consistent with the standard of care for these organisms.

De-escalation included any decrease in generation of cephalosporin, switch from the third-generation cephalosporin ceftazidime to the third-generation cephalosporin ceftriaxone (which has a narrower spectrum of activity among gram negative bacteria), or switch from a carbapenem to any cephalosporin or BL/BLI. Switch to a fluoroquinolone or trimethoprim-sulfamethoxazole (TMP-SMX) from any beta-lactam was considered de-escalation if the cultured organism was susceptible to the empiric beta-lactam agent. If the organism was resistant to the empiric beta-lactam agent used and susceptible to the fluoroquinolone or TMP-SMX, the switch was classified as escalation.

Switch from an intravenous to oral third-generation cephalosporin was considered no change since the switch was made between agents of identical spectrum and might reasonably have been made without formally verifying susceptibility to the oral agent. Cultures were excluded from the analysis if there were other culture data to guide therapy (for example, concurrent treatment for another organism with more extensive antimicrobial resistance), if the patient’s death or discharge to hospice precluded changes to antimicrobial therapy, if AST revealed an opportunity to de-escalate therapy but the managing team did not, if the patient left against medical advice prior to a change in therapy and if the patient was not admitted to the hospital at the time of diagnosis of the bloodstream infection.

Table S3. Classification of all cultures

Value	Count	Percent
No change	14	17.1
Exclude	17	20.7
De-escalation	32	39.0
Escalation	19	23.2

Table S4. Reasons for exclusion of 17 cultures from impact analysis

Value	Count	Percent
Data from another culture specimen influencing antimicrobial choice	6	35.3
Patient death or discharge to hospice	5	29.4
Failure of clinical team to narrow appropriately	3	17.6
Outpatient diagnosis of BSI	2	11.8
Patient left against medical advice	1	5.9

Table S5. Changes considered escalation

Value	Count	Percent
Piperacillin-Tazobactam to Meropenem	5	26.3
Cefepime to Meropenem	4	21.1
Piperacillin-Tazobactam to Cefepime	2	10.5
Ceftazidime to Levofloxacin	2	10.5
Ceftazidime to Cefepime	1	5.3
Ceftazidime to Meropenem	1	5.3
Levofloxacin to Cefepime	1	5.3
Ceftriaxone to Cefepime	1	5.3
Ceftriaxone to Meropenem	1	5.3
Meropenem to Ceftazidime-Avibactam	1	5.3

Table S6. Changes considered de-escalation

Value	Count	Percent
Ceftazidime to Ceftriaxone	5	15.6
Meropenem to Ceftriaxone	4	12.5
Piperacillin-Tazobactam to Ceftriaxone	4	12.5
Cefepime to Ceftriaxone	4	12.5
Meropenem to Levofloxacin	3	9.4
Ceftriaxone to Levofloxacin	3	9.4
Ceftazidime to Levofloxacin	2	6.3
Cefepime to Levofloxacin	2	6.3
Cefepime to Ceftazidime	1	3.1
Meropenem to Ceftazidime	1	3.1
Meropenem to Piperacillin-Tazobactam	1	3.1
Cefepime to Trimethoprim-Sulfamethoxazole	1	3.1
Piperacillin-Tazobactam to Levofloxacin	1	3.1

Table S7: Considered "no change"

Value	Count	Percent
Ceftriaxone to Ceftriaxone	9	64.3
Ceftriaxone to Cefdinir	1	7.1
Ceftazidime to Ceftazidime	1	7.1
Meropenem to Meropenem	3	21.4

Table S8. Changes made for AmpC organisms

Value	Count	Percent
Cefepime to Meropenem	2	22.2
Piperacillin-Tazobactam to Meropenem	2	22.2
Piperacillin-Tazobactam to Cefepime	1	11.1

Ceftriaxone to Cefdinir	1	11.1
Cefepime to Trimethoprim-Sulfamethoxazole	1	11.1
Ceftriaxone to Cefepime	1	11.1
Ceftriaxone to Meropenem	1	11.1

Table S9. Changes made for ESBL organisms

Value	Count	Percent
Cefepime to Meropenem	1	20
Piperacillin-Tazobactam to Meropenem	2	40
Piperacillin-Tazobactam to Cefepime	1	20
Meropenem to Meropenem	1	20

References

1. US Department of Health and Human Services, Antimicrobial Susceptibility Test (AST) Systems—Class II Special Controls Guidance for Industry and FDA. **2009**.
